# “Smart Knee Implants: An Overview of Current Technologies and Future Possibilities”

**DOI:** 10.1007/s43465-022-00810-5

**Published:** 2022-12-28

**Authors:** Edgars Kelmers, Agata Szuba, Samuel W. King, Jeya Palan, Steven Freear, Hemant G. Pandit, Bernard H. van Duren

**Affiliations:** 1 Institute of Medical and Biological Engineering (iMBE), University of Leeds, Leeds, LS2 9JT UK; 2 Leeds Institute of Rheumatic and Musculoskeletal Medicine (LIRMM), University of Leeds, Chapel Allerton Hospital, Chapeltown Road, Leeds, LS7 4SA UK; 3 Leeds Teaching Hospitals NHS Trust, St. James’s University Hospital, Beckett Street, Leeds, LS9 7TF UK; 4 Medical Technologies Innovation and Knowledge Centre, Institute of Medical and Biological Engineering, University of Leeds, Leeds, LS2 9JT UK; 5 School of Electronic an Electrical Engineering, University of Leeds, Leeds, LS2 9JT UK

**Keywords:** Smart implants, Instrumented implants, Active implants, Sensors, TKR, TKA

## Abstract

**Background:**

This article focuses on clinical implementation of smart knee implants for total knee replacement and the future development of smart implant technology. With the number of total knee replacements undertaken growing worldwide, smart implants incorporating embedded sensor technology offer opportunity to improve post-operative recovery, reducing implant failure rates, and increasing overall patient satisfaction.

**Methods:**

A literature review on smart implants, historical prototypes, current clinically available smart implants, and the future potential for conventional implant instrumentation with embedded sensors and electronics was undertaken.

**Results:**

The overview of current and future technology describes use cases for various diagnostic and therapeutic treatment solutions.

**Conclusion:**

Smart knee implants are at an early development stage, with the first generation of smart implants being available to patients and with more novel technologies under development.

## Introduction

Total knee replacement (TKR) is the treatment of choice for patients with severe osteoarthritis and significant symptoms. Over 100,000 TKRs are performed every year in the UK [1] and more than one million globally [2] with up to 143% growth by year 2050 predicted by some authors [3–5]. However, despite this success, a significant number of patients are not completely satisfied. Many patients are reported having ongoing pain (33%), difficulties with rising from a chair (31%), getting out of a car (38%), or climbing stairs (54%) after TKR [6]. With improved life expectancy and increasing preference towards leading an active life, TKRs are increasingly performed in younger and active patients [7]. The Swedish Knee Arthroplasty Register reported that in 2019 TKR use in patients younger than 65 was 7.6 times higher compared to 1993 [8]. According to the data from National Joint Registry of England and Wales in 2019, 2% of primary TKRs were performed on patients aged below 50 and 13% on patients aged between 50 and 59 years [9]. This shift in patient profile results in greater demands put on the replaced joint for longer durations. Such increased demand on TKR implants come with a significant risk for implant failure within a patient`s lifetime. According to the 2021 Australian joint registry data 16.7% of patients under the age of 55 experience primary TKR failure within the first 20 years [10].

Research and development of TKR worldwide is focused primarily on improving patient outcomes and reducing implant failure. To this end, in recent decades, there has been a push towards obtaining data to drive progress. Multiple national joint registries [11] and implant specific registries such as the Orthopaedic Data Evaluation Panel (ODEP) [12] have been implemented. However, these registries offer limited information in that they often only quantify data on implant survival and how implants failed; however, no information is provided on implant performance or implant parameters whilst in active use. Patient Reported Outcome Measures (PROMs) [13] provide a means of gauging patient satisfaction, but again are unable to provide information on implant performance in vivo. As it stands, there are no standards or widely accepted techniques for collecting quantitative data on knee implant performance in vivo [14].

There remains a need to improve recovery after surgery, increase implant longevity, detect failure early, reduce hospital visits, and increase overall satisfaction. Smart implants, with embedded sensor technologies have the potential to provide the quantitative in vivo data to facilitate these goals. The development of smart sensors is faced with many challenges in respect to the materials used, sensor technology, energy sources, size limitation, and data transmission. The incorporated materials need to be biocompatible or isolated from tissue and tailored so that they can be accommodated within the confines of an implant whilst maintaining their function. Moreover, they need to last the lifetime of an implant. Although smart knee implant technology is in its relative infancy there has been significant progress since the first documented implementation in the mid 1990’s [15] up until the recent introduction of a smart knee implant in the commercial market [16]. Implantable sensors have been used to improve understanding of human body, e.g., by measuring forces, torques, pressure, gait kinematics, temperature, and body fluid chemistry [16–20]. The potential benefit of such sensors is the ability to obtain real-life in vivo measurements. Therefore, the sensor technology provides unexplored opportunity to follow-up on patient recovery and implant performance years after the surgery with the help of telemedicine.

This review explores the development and use of smart knee implant technologies to the present date with a focus on their future potential.

In clarification, we use the term smart implants as in reference to implants where electronic or other technology is incorporated within the implant components facilitating measurements or interventions in vivo. Smart implants are not to be confused with wearable sensors (e.g., TracPatch [21] which are only worn on the skin or clothes) and smart orthopaedic instruments (e.g., Verasense trial insert [22] which are only used intraoperatively).

## Past

**Fig. 1:**
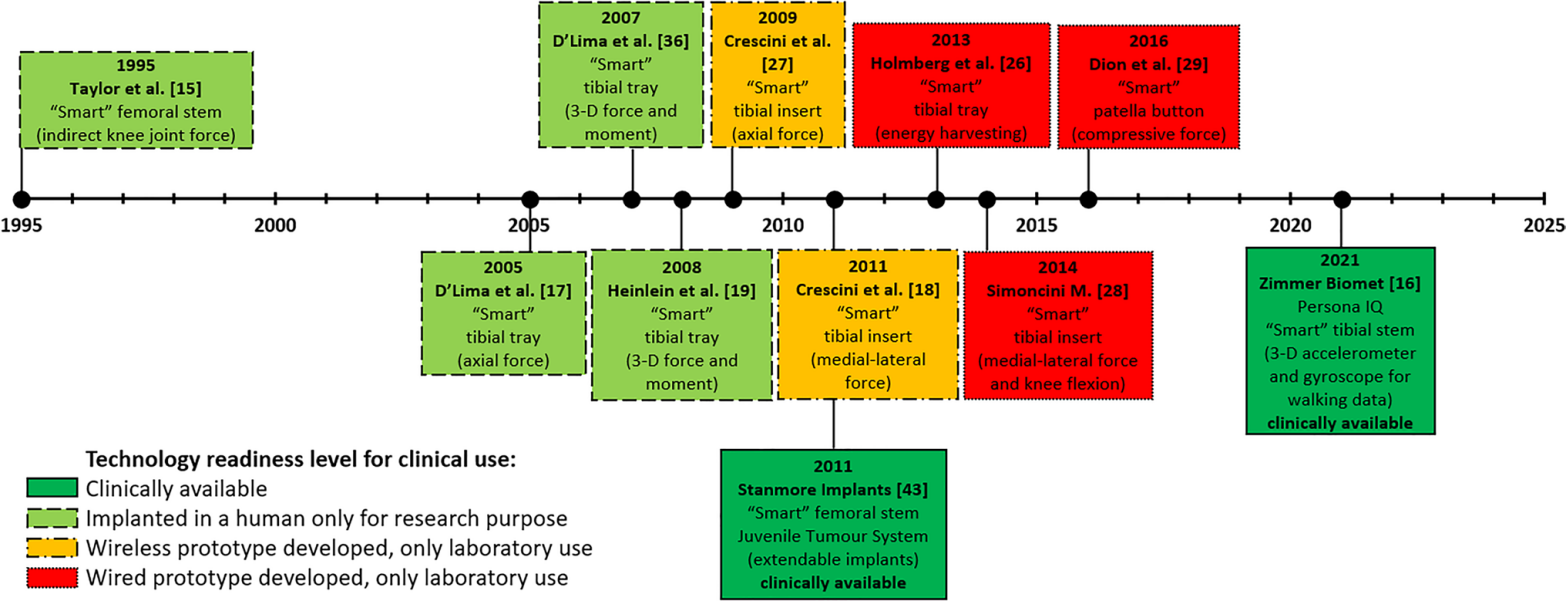
The figure shows a historical timeline to illustrate the development trajectory of smart knee implants

Numerous prototypes of smart knee implants have been in development since 1995 for research purposes [15, 23], however, few of these prototypes have subsequently seen use in vivo. Modern knee implants use a polyethylene insert which is sandwiched between the femoral and tibial components (usually metal alloy, alternates being PEEK and ceramic) in theory providing options for inclusion in or attachment to one or more components. In the literature, to date, instrumentation designs have been described for a distal femoral replacement [24], the tibial tray [17, 25, 26], the polyethylene tibial insert [18, 27, 28], and the patella resurfacing [29]. The historical timeline of the most advanced smart knee implant prototypes is shown in Fig. 1.

The first orthopaedic device that would qualify as a smart implant was an instrumented hip endoprosthesis reported by Rydell in 1966 [30]. Work on the concept of useable telemetric implant designs continued in the 1980’s and 1990’s [24, 31, 32] with focus on hip and proximal femoral endoprostheses. The first wireless smart knee implant was a telemetric distal femoral replacement described by Taylor et al. which was implanted in 1995 in two subjects [15, 33]. Sensors and electronics were placed inside a long titanium 6Al-4 V rod with a drilled hole. The electric power was supplied by an external inductive coupling coil worn around the leg. The same coupling coil was also used for wireless communication. The femoral replacement could measure longitudinal axial force and torque, and two bending moments in the shaft allowing for indirect estimation of knee joint forces.

Subsequent work in the late 1990’s and first decade of the twenty-first century focussed on instrumentation of the tibial component of the knee. Kaufman et al. [23] described an instrumented tibial tray design using four uniaxial load cells sandwiched between a standard tibial component and an additional tray to accommodate the insert. Further development of this concept led to Morris et al. describing the ‘e-Knee’ in 2001 [34] a total knee endoprosthesis incorporating load cells, a micro-transmitter, and an antenna located in the stem of the endoprosthesis and was powered by an external inductive coil. This design was successfully implanted in a single patient and the results reported in 2005 [17]. Subsequently this group upgraded their design [35, 36] to use 12 strain gauges to measure strain in the stem of the tibial tray and determine forces and moments acting on the tray as opposed to first-generation device which used four load cells, one in each quadrant of the tibial tray. This second-generation device was successfully implanted in three subjects and used to evaluate forces generated in a range of activities such as walking, jogging, cycling, tennis, and golf [37]. Around a similar time, Heinlein et al. also developed an instrumented tibial component [19, 38] capable of measuring the six load components (three forces and three moments) being transmitted from the femur to the tibia. Heinlein’s design was implanted in nine subjects and has formed the basis of the TKR component of the OrthoLoad data base [39]. OrthoLoad is an open-access data base sharing kinematics and in vivo loads for knee, hip, shoulder, and spine implants. OrthoLoad data was also used to develop an international testing standard ASTM F3141 [40], which gives standardised loading profiles for daily activities such as walking, turning around, stair climbing, and sitting. Other authors have explored alternative technologies such as piezoelectric energy harvesting [26] and triboelectric transducers to simultaneously sense force and harvest energy [41] incorporated into the tibial component. However, these have not progressed beyond the laboratory.

Some challenges to develop smart tibial and femoral metal components include large dimensions requiring additional bone resection, electromagnetic signal shielding, limited clinical use cases, and high manufacturing costs. To address these, additional work has been reported on developing smart polyethylene inserts. Mentink et al. developed a capacitative sensor to be embedded during the compression moulding process used to manufacture Oxford unicompartmental knee inserts [42]. Simoncini M. [28] reported a smart tibial insert for use in primary surgeries. A wired prototype was developed, allowing to measure knee flexion angle and force under the condyles, however, a wireless prototype has not been reported. Crescini et al. [18, 27] developed two fully enclosed wireless tibial insert prototypes. The first prototype proved the potential to embed the electronics within the ultra-high-molecular-weight polyethylene (UHMWPE) tibial insert while still retaining the original implant dimensions; this prototype used magnetoresistive sensors, which could measure load only up to 12 kg in a single axis [27]. The second prototype used a set of three magnetoresistors to distinguish medial and lateral compression force up to 3000 N [18]. To date, these are the only known wireless smart tibial inserts; however, these designs, as is the case with other instrumented tibial insert designs, have not been implanted.

Instrumentation of the patella button is theoretically also possible. Dion et al. described a smart patella design making use of a passive, wireless force sensor [29]. However, to our knowledge, the use of instrumented patella buttons has not progressed beyond laboratory testing.

## Present

Only a few of the historical designs described have been used in vivo and in these cases for research purposes only. In today’s market, there are only two clinically available smart knee implants: the limb salvage extendable implants developed by Stanmore Implants [43] and the Persona IQ TKR revision implant co-developed by Zimmer Biomet and Canary Medical [16].

In 2011, Stanmore Implants was the first to commercialize Juvenile Tumour System (JTS) extendable implant. The Stanmore extendable implants do not contain any electronics but have an internal mechanism which allows implant extension in vivo [43]. The extendable implant has a telescopic rod connected to magnetically driven gear system which is activated by an external magnetic field. It follows that this implant lengthening technology is limited to use for patients requiring limb lengthening.

In 2021, Zimmer Biomet introduced the Persona IQ smart knee implant with embedded sensors and a telemetry system [44]. It utilises an extended tibial stem with an embedded 3-D accelerometer, a gyroscope, and telemetry system with a claimed battery lifetime for at least 10 years [16, 45]. However, Persona IQ only provides periodic measurements. In the first year after surgery, it collects data from day 2 to day 365, in the second year, only 36 consecutive days per quarter, and afterwards only 36 consecutive days annually [46], which over 10 years’ time sums up to an approximate battery use of 2 years and 2 months. The data from the sensors are used to measure step count, average walking speed, stride length, distance travelled, and tibial range of motion [45].

As the first commercially available smart TKR with embedded sensors, the Persona IQ is a novel device representing a significant step in the progress of smart implant technology. However, this device raises several questions to be addressed in future iterations of smart implants. Due to spatial constraints, the electronics are housed in a tibial stem extension and space for a 58 mm long tibial stem extension [45] is required. This means additional bone resection is required and often an alteration to the implant orientation (tibial slope) is necessary. These factors may also limit the Persona IQ’s use in smaller patients.

To date, there is no evidence of clinical benefit provided by Persona IQ [45] and the argument remains whether similar data can be obtained using wearable sensor technology, e.g., TracPatch [21] can be connected to the mobile phone via Bluetooth and record knee flexion angle, step count, and skin temperature [47].

## Future

Several challenges remain to be addressed which to date have prevented the wider use of instrumented implants. These challenges can be classed as technological, clinical, and ethical. The technology required to achieve practical smart implants presents a number of challenges, the main challenge being to fit into the limited space available within an implant. Implants are designed to minimise the bone resection required and as such there is very little volumetric space to accommodate the electronic components required. This is made even more complex when the implant’s structural integrity must be taken into account. The development of smart implants is a complex, time consuming, and expensive process when design, manufacture, testing, and regulatory approval are considered. BoneTag was created in 2014 and within 8 years, it has only developed two design proofs of concept, but has not resolved yet its manufacturing processes [48]. The Persona IQ product development process took at least 7 years, starting from the first provisional patent application in 2014 [49] until performing the first surgery in 2021 [44], and required investments in total of $46 million from various venture capitals [50, 51]. It has been estimated that up to 7 years are required to develop a conventional medical device from concept to market [52], whilst the current examples indicate that it could take even longer for smart implant development. Clinically, the use of smart implants remains a challenge as their clinical effectiveness remains to be proven. This is a challenge as only a handful of such implants have been tried to date and requires extensive controlled testing once the technological hurdles have been overcome.

The use of sensors in medical technology does come with some ethical considerations. Patient data ownership and who should/could it be shared with, remains an ongoing discussion. The potential data from smart implants leads to competing interests. On one hand, getting large data sets to gain a better understanding of trends, what is normal and what is abnormal in terms of patient outcomes, will only be possible if such data is shared. On the other hand, although the public knowingly (or inadvertently) share a lot of personal data with third party (e.g., through social media, retail on-line services), they may be reluctant to do the same for meaningful clinical information to be shared with other clinicians/researchers/policy makers. Given these considerations, work remains to be done on how this potentially very large amount of data will be managed and regulated. At this moment TracPatch and Persona IQ products have resolved the data anonymization question by recording only kinematic data [21, 46]. To our knowledge, no patient sensitive personal data, such as, name, address, medical images and records, or GPS tracking are stored on the medical device itself [46, 53]. Furthermore, Persona IQ protects mobile data by following HIPPA security processes [46]. Even so, TracPatch disclaims that 100% mobile device security is not guaranteed [53].

Although smart implants have seen limited clinical use to date there has clearly been a notable growing trend of smart implant technology development. This is evidenced by the rapid growth in number of smart implant technology related patents. A patent search in software Orbit Intelligence (Questel, France) [54] comprising the keywords (implant or prosthe + and sensor + and joint or knee and wireless) revealed that the first five patent applications were made in 2001, and in the time period between 2001 and 2021 in total over 300 patent applications have been filled in. This shows an increasing industry interest in smart implant development. Similarly, whilst the current smart implants have limited clinical influence, growing research is being undertaken both by universities and private companies to further improve relevant technologies targeting numerous applications:

### Implant Identification and Patient Data

Future implants will benefit from having an internal memory, which would allow for implant and relevant medical data to be stored [55]. In addition to the functionality of data storage, integrated memory would improve power consumption profile when compared with continuous wireless data transfer.

### Measuring Load and Range of Motion

Measurement of load and motion give quantitative information about implant performance and patient recovery. Combined pressure and motion measurements could indicate if the implant is overloaded [56] or load measurements could be related to implant wear, deformation, and stress distribution within the implant or the periprosthetic bone [23]. Smart implants like Persona IQ could use motion data to track recovery, for example, whether the user is improving their range of motion, increased walking speed, or walking distance. The company BoneTag [48] is working on technology to allow temperature, force, and kinematic measurements.

### Adjustable Implants In Vivo

Potentially implant alignment could be adjusted in vivo. Conceivably, micro-motors combined with an actuation mechanism could be placed inside the tibial tray allowing to change the height of the medial or lateral tibial condyle [57]. Successful implementation would allow implants to monitor and act on any ligament imbalance after surgery.

### Detection of Implant Loosening

Loosening is a frequent cause for TKR failure [1, 10]. Early detection of implant loosening, or bone resorption could be achieved by measuring temperature around the implant [58], by using mechanical magnetic sensors with an oscillating membrane [59] or with an embedded ultrasound system [60].

### Monitoring Wear

With increasing numbers of younger patients undergoing TKR, the consequences of wear are likely to become more prominent. Early detecting and ongoing monitoring of wear could facilitate liner exchange prior to wear induced aseptic loosening. To provide early diagnosis, tibial insert wear could be indirectly estimated by measuring UHMWPE thickness with capacitive sensors [61], optical sensors, or ultrasonic-based sensors [62]. Alternatively, component wear could potentially be predicted with biosensors by analysing synovial fluid for particles of metal [63], UHMWPE, bone, or cement [64].

### Detecting and Treating Infection

Early diagnosis of infection is an attractive proposition given the potential benefits of early detection and treatment. Several sensor technologies have been explored to facilitate early infection detection. The simplest approach is to measure the temperature of implant components or the surrounding tissue [60, 65]. More advanced approaches involve the analysis of bodily fluids with biosensors [20]. For example, Profusa [66] is developing injectable body sensors to monitor internal body chemistry, however, currently, this is limited to glucose measurement [67]. Implants with a magnetoelastic coating could help prevent/treat infection. Theoretically, an external AC magnetic field could be used to vibrate the magnetoelastic coating, which would prevent or dislodge cell on-growth on implant surfaces allowing for increased effectiveness of antibiotics [68]. Integrated drug delivery systems could be considered for targeted and controlled in vivo drug release [69–71]. Drug-eluting implants have been described with fixation pins [72] and screws [73]. ForCast Orthopedics [74] describe an actuated implantable drug delivery system for localized delivery of antibiotics to treat endoprosthetic joint infections.

### Power Supply

Sensors are often designed to last for the entire life-span of an implant, whilst batteries have a finite life [75]. Two options exist for powering sensors: battery power and external power [25]. Battery powered smart implants provide an effortless measurement experience over long time and during dynamic activities, but the drawback is large dimensions and limited battery charge. Therefore, further research is conducted to reduce electronic component size or enable self-powering with piezoelectric or kinetic energy power harvesting implants [25, 75]. Externally powered sensors are cheaper and more versatile, since due to the small dimensions they could be embedded in any implant component and last for a lifetime [75]. The disadvantage, however, is that an external power supply must be worn during the measurements, which restricts the practical use mostly to measurements in laboratory conditions. Alternatively, an external battery pack must be worn to take measurements outdoors [76].

## Conclusion

Ultimately, it can be surmised that smart implant technology in knee replacement is in its infancy; particularly if we consider successful clinical application, which is limited to the extendable distal femoral replacement by Stanmore Implants [43] and the recent introduction of Zimmer Biomet’s Persona IQ [44]. A significant increased interest has been shown over the past 2 decades which has led to a wide range of innovative ideas; several of which we have summarised here. Despite this growing interest, we expect it will be decades before much of the technology we have discussed here will reach clinical implementation and validation. Nevertheless, these are exciting times in which we expect to see rapid progress in relevant technologies across the smart implants field and look forward to seeing the realisation of the potential clinical benefits for our patients.

## Data Availability

No data are associated with this article.
